# Strain-level genomic analysis of serotype, genotype and virulence gene composition of group B streptococcus

**DOI:** 10.3389/fcimb.2024.1396762

**Published:** 2024-11-06

**Authors:** Zhen Zeng, Meng Li, Simin Zhu, Ke Zhang, Yifan Wu, Minzi Zheng, Yang Cao, Zhenyu Huang, Qinping Liao, Lei Zhang

**Affiliations:** ^1^ Department of Obstetrics and Gynecology, Beijing Tsinghua Changgung Hospital, School of Clinical Medicine, Tsinghua University, Beijing, China; ^2^ Institute for Precision Medicine, Tsinghua University, Beijing, China

**Keywords:** group B *streptococcus* (GBS), surveillance, genotype, serotype, virulence gene

## Abstract

**Introduction:**

GBS (group B streptococcus) is an opportunistic pathogen that can colonize healthy individuals but presents significant challenges in clinical obstetrics and gynecology, as it can cause miscarriage, preterm birth, and invasive infections in newborns. To develop specific and personalized preventative strategies, a better understanding of the epidemiological characteristics and pathogenic features of GBS is essential.

**Methods:**

We conducted a comprehensive strain-level genomic analysis of GBS, examining serotype and genotype distributions, as well as the composition and correlations of virulence genes using the blastn-short mode of the BLAST program(v2.10.0+), mlstsoftware (https://github.com/tseemann/mlst), Snippy (v4.6.0), FastTree (v2.1.11) and iTOL. The coding sequence region of virulence factors was annotated by Prodigal (v2.6.3) and Glimmer(v3.02b). We further identified host protein interacting with Srr2 by mass spectrometry analysis.

**Results:**

While certain genotypes showed strong serotype consistency, there was no significant association between overall serotypes and genotypes. However, the composition of virulence genes was more closely related to the phylogeny of GBS, among which simultaneous presence of Srr2 and HygA exhibit significant association with hypervirulence. Tubulin emerged as the most distinct and abundant hit. The specific interaction of Tubulin with Srr2-BR, rather than Srr1-BR, was further confirmed by immunoblotting.

**Discussion:**

Considering the impact of cytoskeleton rearrangement on GBS pathogenesis, this observation offers a plausible explanation for the hypervirulence triggered by Srr2. Collectively, our findings indicate that in the future clinical practice, virulence gene detection should be given more attention to achieve precise GBS surveillance and disease prevention.

## 1 Introduction

*Streptococcus agalactiae*, commonly referred to as Lancefield group B *streptococcus* (GBS), is a Gram-positive coccus that was initially recognized as a causative agent of bovine mastitis. It is commonly found in the gastrointestinal and genital tracts of healthy individuals. GBS can cause various infections, particularly in newborns, pregnant women, the elderly, and immunocompromised individuals (Verani et al., 2010). During pregnancy and childbirth, it is important to consider the presence of GBS in the vaginal and rectal regions, as it can increase the risk of ascending infection, potentially leading to preterm birth and stillbirth (Hillier et al., 1991; Allen et al., 1999). Furthermore, GBS may cause serious infections such as neonatal sepsis, pneumonia, and meningitis through mother-to-child transmission during delivery. GBS is also implicated in a number of other infections, including soft-tissue and urinary tract infections, arthritis, sepsis, endometritis and aerobic vaginitis (Jackson et al., 1995; Krohn et al., 1999; Zeng et al., 2023).

GBS typing can elucidate differences in pathogenicity, antibiotic resistance, and genetic diversity among strains, facilitating epidemiological surveillance and the development of more effective interventions (Furfaro et al., 2018). Based on the composition of capsular polysaccharide, a total of 10 serotypes, namely Ia, Ib, and II-IX, have been identified (Cieslewicz et al., 2005). Approximately 98% of the serotypes identified during maternal colonization and 97% of those identified during neonatal disease are estimated to be encompassed by six serotypes (Ia, Ib, II, III, IV and V). Among them, serotype-III is relatively clearly associated with invasive disease in newborns while serotypesIa and V are the dominant invasive isolates in non-pregnant cases (Alhhazmi et al., 2016). The prevalence of serotypes in maternal colonization or neonatal disease varies significantly by regions on a global scale and shows no clear pattern (Madrid et al., 2017). With multilocus sequence typing (MLST), GBS can be classified into more than five hundred of sequence types (STs) and then can be clustered into clonal complexes (CCs) (Furfaro et al., 2018). The most prevalent STs include ST-17, ST-1, ST-23, ST-19, ST-10, ST-8, ST-484, ST-182, ST-28, ST-12, ST-24, and ST-7, which corresponds 5 CCs (CC-17, CC-1, CC-23, CC-19, CC-10) (Furfaro et al., 2018). ST-1, ST-17, and ST-19 were reported to be more frequently associated with invasive neonatal disease (Bisharat et al., 2005; Kang et al., 2017).

A series of virulence factors are encoded by GBS to facilitate vaginal colonization and the occurrence of ascending infection. These virulence factors mainly function in adhesion, invasion, immune evasion, and environmental adaptation (Vornhagen et al., 2017). Adherence and invasion can be mediated by interactions between surface adhesin protein of GBS and host cell and extracellular matrix components (ECMs) (Landwehr-Kenzel and Henneke, 2014; Shabayek and Spellerberg, 2018). Functionally characterized adhesins that mediate GBS adhesion and/or host invasion include fibrinogen binding protein (Fbs), laminin binding protein (Lmb), group B streptococcal C5a peptidase (ScpB), streptococcal fibronectin protein binding protein A (SfbA), GBS immunogenic bacterial adhesin (BibA), host cell surface glycosaminoglycan binding protein (Alpha C protein), and pili (Shabayek and Spellerberg, 2018). These interactions may also promote GBS resistance to mechanical clearance, avoidance of immune surveillance, and paracellular transmigration. In addition to adhesion-related virulence factors, hyaluronidase (HylB), hemolysin, and capsule play significant roles as virulence factors in GBS. These factors have been reported to contribute to GBS colonization in the vagina, ascending infection, immune evasion, placental invasion, and fetal injury (Whidbey et al., 2013; Kolar et al., 2015; Vornhagen et al., 2016).

Currently, the primary clinical strategy against GBS involves universal culture-based screening for maternal colonization at 35 to 37 weeks of gestation and prophylactic antibiotic administration (Prevention of perinatal group B streptococcal disease: a public health perspective. Centers for Disease Control and Prevention, 1996; Schrag et al., 2002; Prevention of group B streptococcal early-onset disease in newborns: ACOG committee opinion, number 782, 2019). There are also studies exploring the use of nucleic acid amplification tests (NAAT) for GBS screening. Compared to culture methods, NAAT offers comparable sensitivity and specificity. However, concerns remain about its high cost and inability to perform antibiotic susceptibility testing. Consequently, NAAT has not been widely adopted or validated in clinical practice (Church et al., 2017). With current strategies to screen for GBS colonization status, the incidence of neonatal GBS early-onset disease (EOD) was reduced to significantly. However, the biological characteristics of GBS infection are not well characterized by this approach, and the relationship between GBS and normal colonization and infection is not well established (Vornhagen et al., 2017). Currently, the comprehensive burden of GBS infection, including GBS late-onset disease (LOD), is not fully addressed. Furthermore, intrapartum antibiotic prophylaxis faces challenges such as antibiotic resistance and the increased incidence of non-GBS neonatal pathogens (Castor et al., 2008).

In this study, we analyzed the composition of GBS virulence genes and their correlation with various GBS genotyping methods on a global scale. Our goal is to provide references for clinical monitoring and disease prevention of GBS, as well as uncover insights into the pathogenesis and process of GBS. Through the analysis of GBS virulence genes, we summarized the distribution of different virulence genes in GBS, and 78% of the 1,552 genomes were categorized into 14 groups. We found that the distribution of virulence genes in GBS serotypesIII and ST-17 was obviously homogeneous, and compared with non-human GBS, especially fish-derived GBS, human GBS had more virulence genes. Besides, to augment the biological evidence for current study, we purified the binding regions of Srr1 and Srr2, and compared their host interactors by mass spectrometry analysis. Significantly, the microtubule cytoskeleton protein Tubulin was found to specifically interact with Srr2-BR, suggesting that the observed binding affinity of Tubulin may provide a mechanistic explanation for the specific induction of hypervirulence by Srr2-BR. Taken together, this information holds potential benefits in clinical settings, providing valuable insights into the characteristics of GBS infection and informing disease prevention strategies.

## 2 Materials and methods

### 2.1 Genomic information collection

Genome sequences was collected from the NCBI Reference Sequence Database. The average nucleotide identity (ANI) to type strains (*S. agalactiae* NCTC 8181, GCF_900458965.1) was analyzed using fastANI (v1.33) to confirm the taxonomic status of the genomes (Jain et al., 2018). The quality of genome assemblies was evaluated using BUSCO (v5.4.7) (Manni et al., 2021).

### 2.2 Serotyping, genotyping and phylogenetic analysis

Refer to the previous study of Breeding et al. to obtain the specific target sequences of different GBS serotypes(Ia, Ib, II, III, IV, V, VI, VII, VIII). Then these sequences were aligned to the genome using the blastn-short mode of the BLAST program(v2.10.0+) for serotyping (Breeding et al., 2016). MLST and single nucleotide polymorphism (SNP) were conducted using mlstsoftware (https://github.com/tseemann/mlst) and Snippy (v4.6.0), respectively (Jolley and Maiden, 2010; Silva et al., 2018). Phylogenetic trees based on SNP and annotations were constructed with FastTree (v2.1.11) and iTOL (Price et al., 2010; Letunic and Bork, 2021).

### 2.3 Prediction of virulence genes

Fifty-eight coding genes associated with fifteen virulence factors were used as reference sequences for downstream analysis (**Table 1**) (Jerlstrom et al., 1996; Bohnsack et al., 1997; Gravekamp et al., 1998; Areschoug et al., 2002; Spellerberg et al., 2002; Baron et al., 2004; Gutekunst et al., 2004; Brown et al., 2005; Pietrocola et al., 2005; Bolduc and Madoff, 2007; Forquin et al., 2007; Pannaraj et al., 2007; Tenenbaum et al., 2007; Carlin et al., 2009; Klinzing et al., 2009; Devi and Ponnuraj, 2010; Tazi et al., 2010; Al Safadi et al., 2011; Sheen et al., 2011; Pezzicoli et al., 2012; Seo et al., 2012; Seo et al., 2013; Buscetta et al., 2014; Chang et al., 2014a; Chang et al., 2014b; Rosa-Fraile et al., 2014; Maeland et al., 2015; Six et al., 2015; Gabrielsen et al., 2017; Benito-Jardon et al., 2020; Dobrut and Brzychczy-Wloch, 2021; Creti et al., 2023). These virulence factors were reported to be related to GBS colonization, immune evasion and invasion. The coding sequence region was annotated by Prodigal (v2.6.3) and Glimmer(v3.02b), respectively (Delcher et al., 2007; Hyatt et al., 2010). The predicted amino acid and nucleic acid sequence were aligned to the reference virulence gene sequence by Diamond (v2.1.9) and Abricate (v1.0.1) (https://github.com/tseemann/abricate) (Buchfink et al., 2015). When comparing the annotation results of virulence genes obtained by different methods, consistent results were directly interpreted as positive. Inconsistent results were manually corrected to provide the final annotation.

**Table 1:** Different virulence factors and their encoding genes.

Virulence factors	Gene	Function/Characteristic	Ref
β-protein(Bac)	*cba*	Bind to the surface sialic acid-binding immunoglobulin-like lectin 5 (Siglec-5) and Siglec-14 of leukocytes, weakening the innate immune response of infected organisms.	(Jerlstrom et al., 1996; Areschoug et al., 2002; Pannaraj et al., 2007; Carlin et al., 2009; Dobrut and Brzychczy-Wloch, 2021)
Laminin binding protein (Lmb)	*lmb*	Mediate the binding and colonization of GBS and laminin.	(Tenenbaum et al., 2007)
Fibrinogen binding protein A	*fbsA*	Promotes GBS attachment to fibrinogen.	(Pietrocola et al., 2005)
Fibrinogen binding protein B	*fbsB*	Promote GBS invasion into epithelial cells.	(Spellerberg et al., 2002; Gutekunst et al., 2004; Devi and Ponnuraj, 2010; Al Safadi et al., 2011)
Fibrinogen binding protein C(Also known as GBS surface adhesion BsaB)	*fbsC*	Promote invasion of epithelial and endothelial barriers	(Buscetta et al., 2014)
Serine rich repeat proteins (Srr)	*srr1*	Binding of glycoprotein Srr1 of GBS to fibrinogen Promotes Attachment to brain endothelium and the development of meningitis.	(Sheen et al., 2011; Seo et al., 2012)
	*srr2*	Srr2 is a multifaceted adhesin used by the ST-17 clone to hijack ligands of the host coagulation system, thereby contributing to bacterial dissemination and invasiveness, and ultimately to meningitis.	(Seo et al., 2013; Six et al., 2015)
Streptococcal peptidase C5a	*scpB*	Inactivates C5a, a product of the complement cascade, weakening neutrophil recruitment.	(Bohnsack et al., 1997; Brown et al., 2005)
Capsular polysaccharides (CPS)	*cps*cluster(*cpsA, cpsB, cpsC, cpsD, cpsE, cpsF, cpsL, neuB, neuC, neuD, neuA, cpsG, cpsK, cpsH, cpsJ, cpsM, cpsN, cpsO*)	Inhibits the binding of the activated complement factor C3b to the surface of GBS, preventing the activation of the alternative complement pathway and inhibits complement-mediated opsonophagocytosis.	(Pezzicoli et al., 2012; Chang et al., 2014a; Chang et al., 2014b)
β-hemolysin/cytolysin	*cyl*cluster (*cylX, cylD, cylG, acpC, cylZ, cylE, cylF, cylL, cylJ, cylK, cylA, cylB*)	Forms pores in cell membrane. Proinflammatory effects: inducing apoptosis, promoting cellular invasion, triggering iNOS and cytokine release.	(Forquin et al., 2007; Rosa-Fraile et al., 2014)
Hyaluronidase	*hylB*	Facilitates spread of bacteria by breaking down the hyalurone polymers present in the extracellular matrices of the host. The GBS hyaluronate lyase also has limited specificity for achondroitin sulphate and cleaves the chain at unsulphated sites. This action may facilitate deep tissue penetration during infection.	(Kolar et al., 2015)
Fibronectin binding protein A(SfbA)	*sfbA*	Invasion of endothelial cells, invasion of vaginal and cervicalepithelial cells	(Shabayek and Spellerberg, 2018)
Hypervirulent GBS adhesin (HvgA)	*hvgA*	Crucial factor for both colonization and invasion by hypervirulent GBS clones, facilitating their ability to adhere to and translocate across epithelial barriers, ultimately leading to meningitis development in neonates.	(Rosa-Fraile et al., 2014)
Pili	PI-1(gbs0628, gbs0629, strC1, strC2, gbs0632); PI-2a(pilA, strC1, strC2, pilB, pilC)	Mediating cell attachment, promoting the invasion of human endothelial cells and may facilitate paracellular translocation across the epithelial barrier.	(Sheen et al., 2011)
Alpha-Like Proteins	*bca, alp1, alp2, alp3, alp4, rib*	Mediates bacterial internalization	(Gravekamp et al., 1998; Baron et al., 2004; Bolduc and Madoff, 2007; Klinzing et al., 2009; Maeland et al., 2015; Gabrielsen et al., 2017; Benito-Jardon et al., 2020)
CAMP factor	*cfb*	Forms pores in cell membrane by oligomerization	(Creti et al., 2023)

### 2.4 NAAT for GBS screening vs. culture methods

Vaginal and anal swabs were collected from 43 pregnant women at 36~37 + 6 weeks of pregnancy, and samples were taken at the same time. After sampling, GBS was detected by NAAT and selective culture medium. NAAT was performed using registered 7500 real-time fluorescence quantitative PCR kits following the relevant instructions, and the CT value is set to less than 35, which is considered as GBS colonization.

### 2.5 Protein expression and purification

DNA fragments spanning the Srr1-BR and Srr2-BR with Flag tag were synthesized by Sangon Biotech (Shanghai, China), and cloned into pET28a expression vectors. Proteins were expressed in E. coli BL21 Chemically Competent Cell (TransGen Biotech, Beijing, CHN). After treated with 0.5 mM IPTG in 16°C overnight, cells were harvested with buffer A (20 mM Tris–HCl and 50 mM NaCl) and clarified by ultrasonication. Lysates were centrifuged at 12,000 g for 30 min in 4°C. Then the supernatant of the lysates was harvested and incubated with Ni2+ Sepharose for 4 h at 4°C. Subsequently, proteins were purified by Ni2+ sepharose, then concentrated by Amicon Ultra-10 at 4°C. Protein concentration was determined using the Bradford assay with BSA as the standard.

### 2.6 Affinity purification and mass spectrometry analysis

To identify Srr1-BR and Srr2-BR-interacting proteins, two of 10 cm dishes HEK293T cells were lysed in 4 ml of ice-cold buffer containing 20 mM Hepes (pH 7.4), 150 mM NaCl, 0.3% Triton-X-100 and Complete Mini protease inhibitors (EDTAfree, Roche) for a duration of 10 minutes. The resulting lysate was subjected to centrifugation at 5,000 × g for 5 minutes to remove nuclei and insoluble cellular components. The supernatant was collected thereafter. Then 10 μg of Srr1-BR or Srr2-BR along with anti-FLAG M2 agarose were then added to the supernatant in 4°C overnight. Following this step, washing procedures were performed using ice-cold buffer containing 20 mM Hepes (pH 7.4), 150 mM NaCl and 0.1% Triton-X-100. Subsequently, Srr1-BR or Srr2-BR-interacting proteins were eluted using 3x FLAG peptide (150 ng/μl).

The protein bands were subjected to in-gel trypsin digestion, followed by extraction of peptides from the gel matrix and resuspension in an aqueous buffer for subsequent LC-MS/MS analysis.

### 2.7 Immunoblotting

The eluted proteins that interacted with Srr1-BR or Srr2-BR were resolved on 12% SDS-PAGE gels. Subsequently, the transferred proteins onto PVDF membranes were incubated with specific antibodies (β-Tubulin: proteintech, 10068-1-AP; Flag: Sigma, F1804) and visualized using the super sensitive ECL luminescence kit (# MA0186-1, Meilunbio, Meilun Biotechnology co. Ltd, Dalian, China).

## 3 Results

### 3.1 Serotypes and genotypes assignment

**Figure 1:**
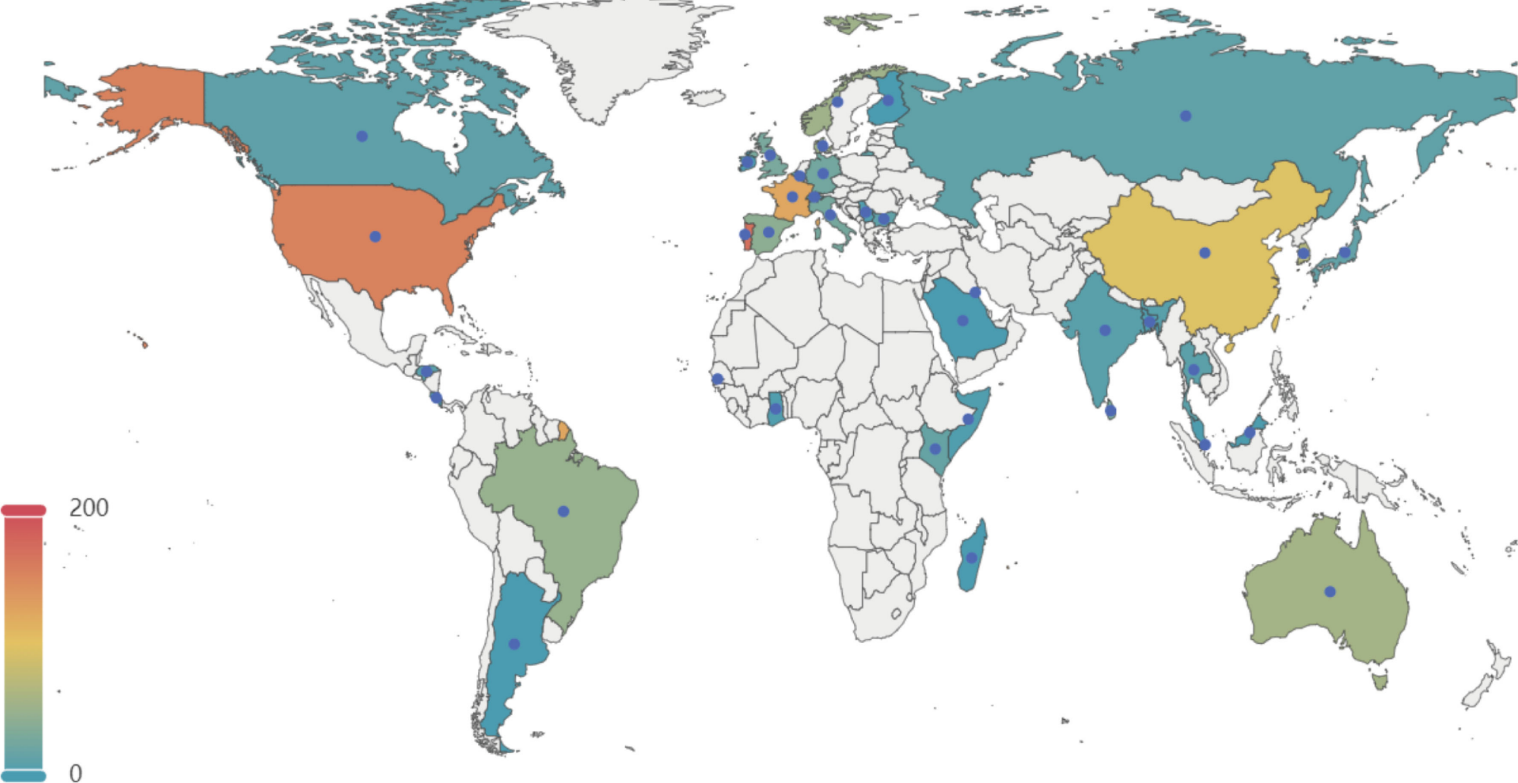
Geographical distribution of 1, 552 GBS strains selected in this study. This map illustrates the global distribution of the 1,552 GBSstrains analyzed in the study. The color gradient represents the number of strains from each country, with warmer colors indicating higher numbers. Key regions of interest include the United States, Portugal, France, China, and Korea, among others. The data points help to visualize the geographical diversity of the GBS strains included in the analysis.

A total of 1,552 genomes with ANI greater than 98% to the reference and genome completeness exceeding 95% were included in the analysis. The GC content of these genomes ranges from 35% to 40%, and their sizes vary between 1.68 Mb and 2.48 Mb. Among these 1,552 genomes, 1,201 GBS strains were isolated from human sources, while 351 genomes originated from non-human sources. The non-human GBS strains were primarily derived from bovine and fish hosts. The primary geographical regions included in this study are Portugal, the United States, France, China, and Korea (**Figure 1**).

**Figure 2:**
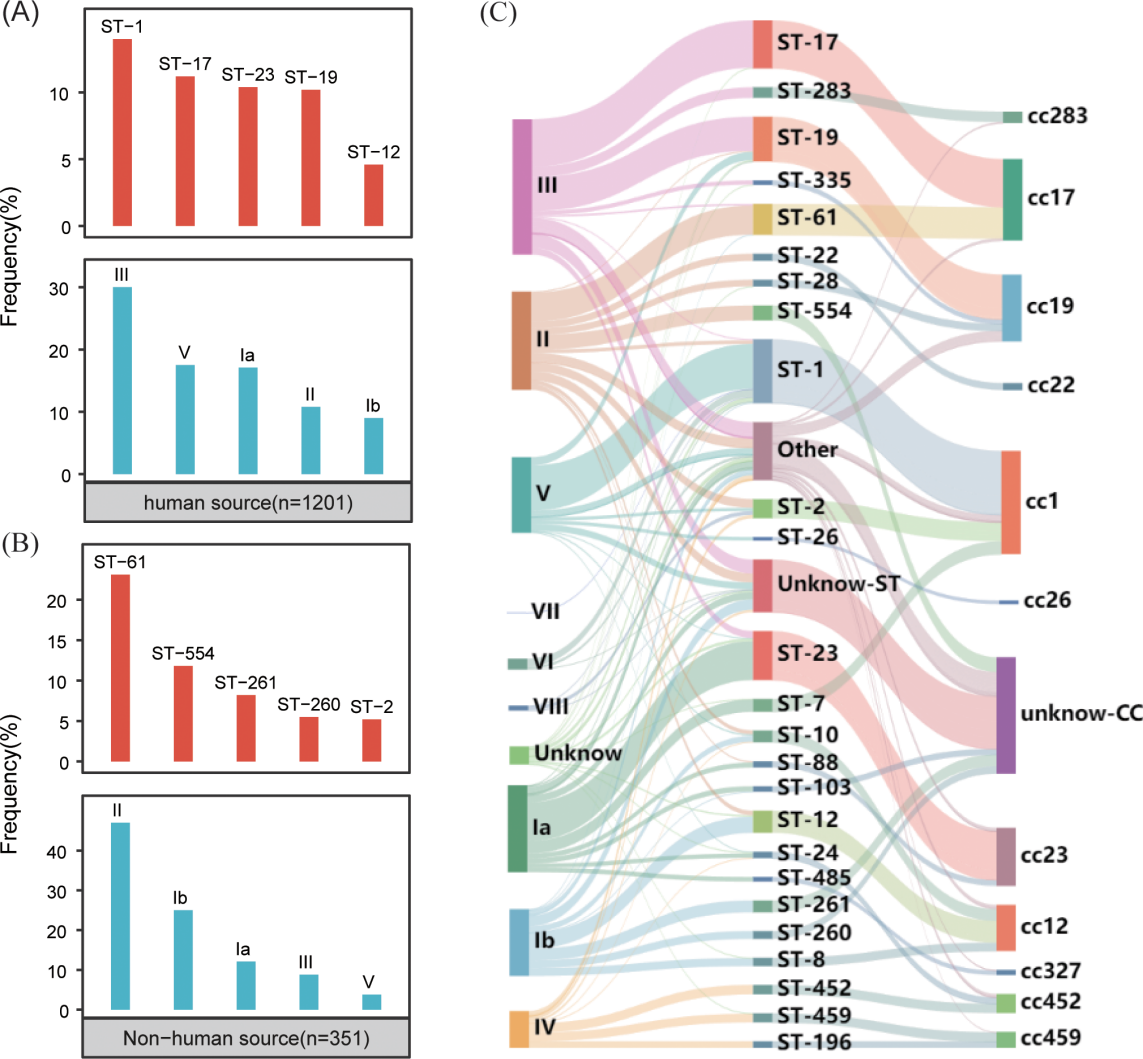
Distribution of GBS serotypes and genotypes. **(A)** The frequency distribution of the top five sequence types (STs) and serotypes in human-source GBS strains (n=1201). **(B)** The frequency distribution of the top five sequence types (STs) and serotypes in non-human-source GBS strains (n=351). **(C)** The correspondence between serotypes and sequence types (STs) in human-source GBS strains, illustrating the relationship between specific serotypes and genotypes.

The typing results revealed that 97% (1,500/1,552) of the genomes were classified into nine distinct serotypes, and 92% (1399/1552) were classified into 115 different genotypes. In the GBS strains collected from human sources, the most common serotypes, ranked from highest to lowest, were III, V, Ia, II, and Ib. The most commonly identified genotypes in these strains were ST-1, ST-17, ST-23, ST-19 and ST-12 (**Figure 2A**). Conversely, the GBS strains from non-human sources most frequently fell into serotypes II, Ib, Ia, III, V and most common genotypes were ST-61, ST-554, ST-261, ST-260, and ST-2 (**Figure 2B**).

The correlation analysis across all GBS serotypes and genotypes reveals that GBS type III is primarily associated with ST-17 and ST-19, collectively making up 35.0% (138/394) and 31.2% (123/394) of the total strains respectively. Type II is predominantly linked with ST-61 and ST-554, contributing to 29.4% (84/286) and 15.0% (43/286) respectively. Serotype Ia is mainly associated with ST-23, constituting 44.7% (113/253). Serotype Ib is largely connected with ST-12 and ST-261, together accounting for 24.2% (47/194) and 17.5% (34/194) respectively. Type V is primarily tied to ST-1, making up 70.9% (156/220). While some MLST genotypes such as ST-17 exhibit significant serotype homogeneity and belong to serotypes III, the overall distribution of serotypes and genotypes shows a cross-relationship, as illustrated in **Figure 2C**.

**Figure 3:**
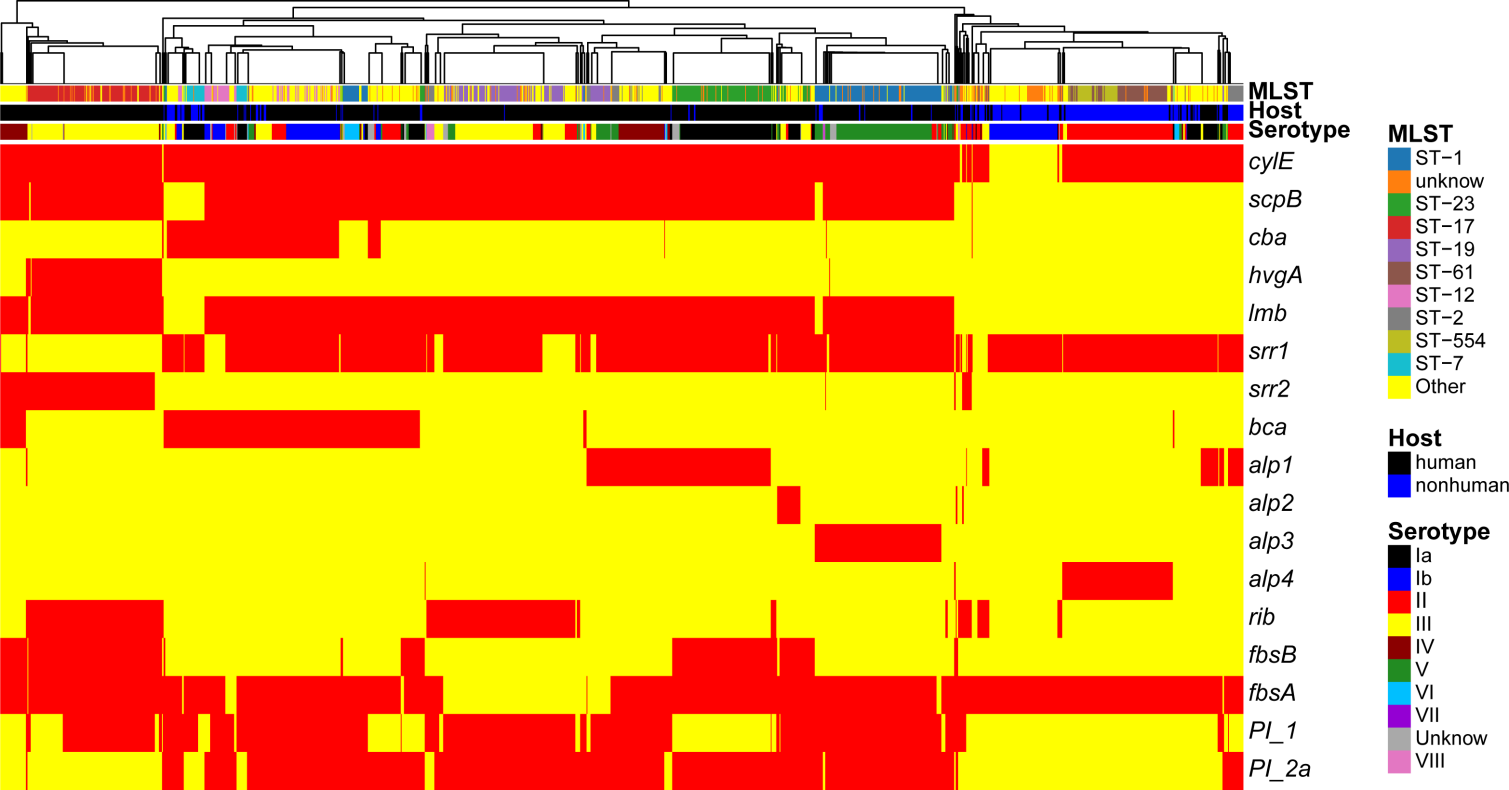
Proportional distribution of different virulence genes in human-source GBS and nonhuman-source GBS. This bar graph displays the frequency (%) of various virulence genes in GBS strains from three sources: all genomes (n=1,552), human-source (n=1,201), and non-human-source (n=351). The virulence genes are listed along the x-axis, and their corresponding frequencies are represented on the y-axis. The different colors indicate the source of the GBS strains: blue for all genomes, orange for human-source, and green for non-human-source.

A phylogenetic analysis based on genome-wide SNPs was performed on all GBS strains. The findings, as illustrated in **Figure 3**, demonstrated that MLST displayed superior phylogenetic consistency in comparison to serotyping. Regarding geographical dispersion, GBS strains isolated from non-human hosts (primarily bovine) in Portugal exhibited a strong correlation with geographical consistency. However, no discernible new patterns within the geographical distribution of GBS were identified.

### 3.2 Virulence genes profiles

**Figure 4:**
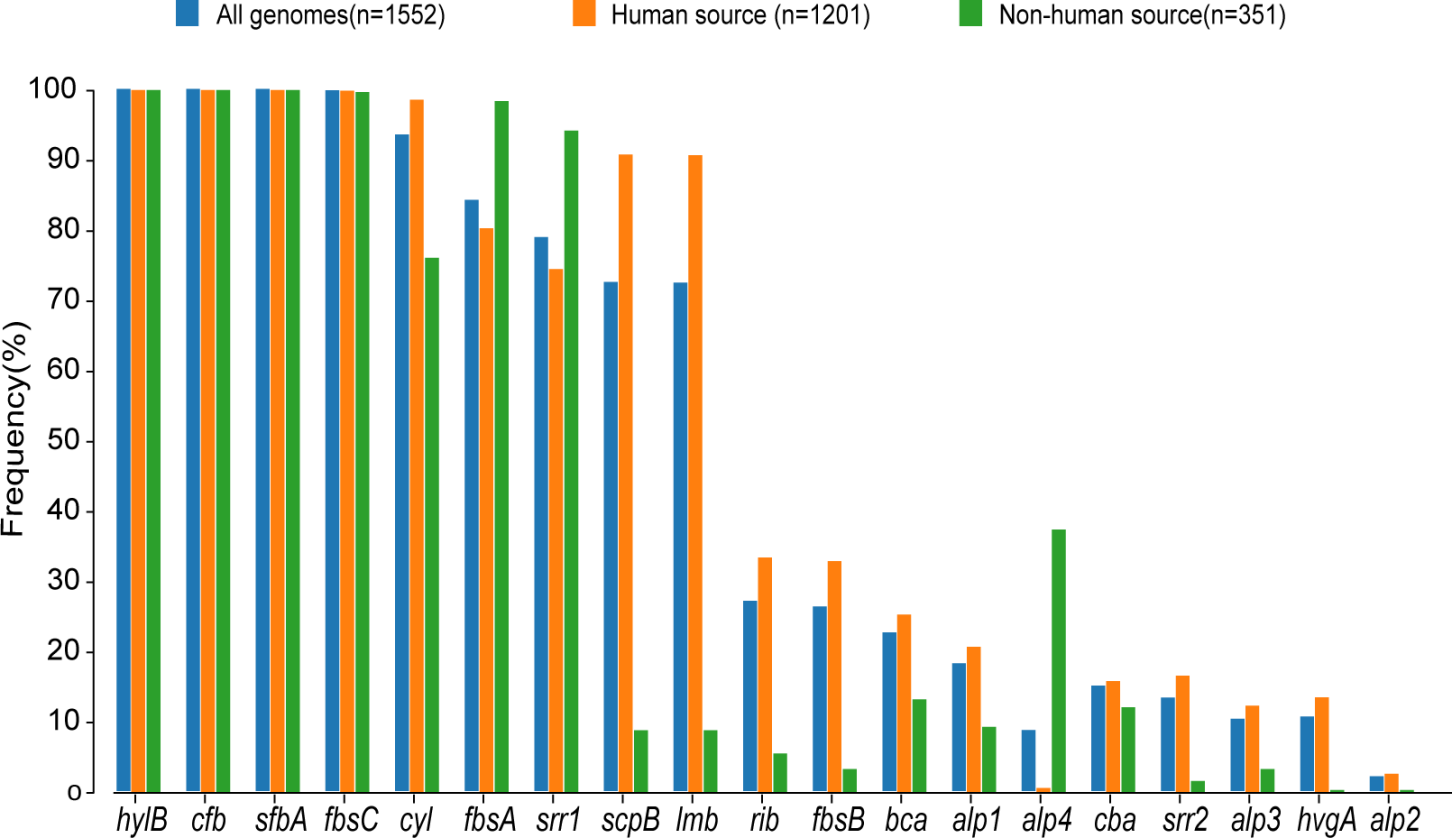
Distribution of CPS gene clusters in different serotypes. This heatmap illustrates the distribution of capsular polysaccharide (CPS) gene clusters across various GBS serotypes. The rows represent different CPS genes, while the columns represent individual GBS strains. The color intensity in each cell reflects the presence (red) or absence (yellow) of a specific CPS gene in a given strain.

The examination of virulence gene composition across 1,552 genomes has demonstrated variations in the distribution proportions of distinct virulence genes within GBS. As depicted in **Figure 4**, the genes *hylB*, *cfb*, *sfbA*,*fbsC*, *cyl* clusters, *fbsA*, *srr1*, *scpB*, and *lmb* were ubiquitously distributed in GBS, with the least prevalent gene still being found in over 70% of cases. Intriguingly, the genes *hylB*, *cfb*, *sfbA*, and *fbsC* were annotated across all genomes analysed. However, a smaller proportion of genomes contained the genes *cba*, *fbsB*, *hvgA*, and *srr2*, with distribution rates ranging from 10.6% to 26.3%.

**Figure 5:**
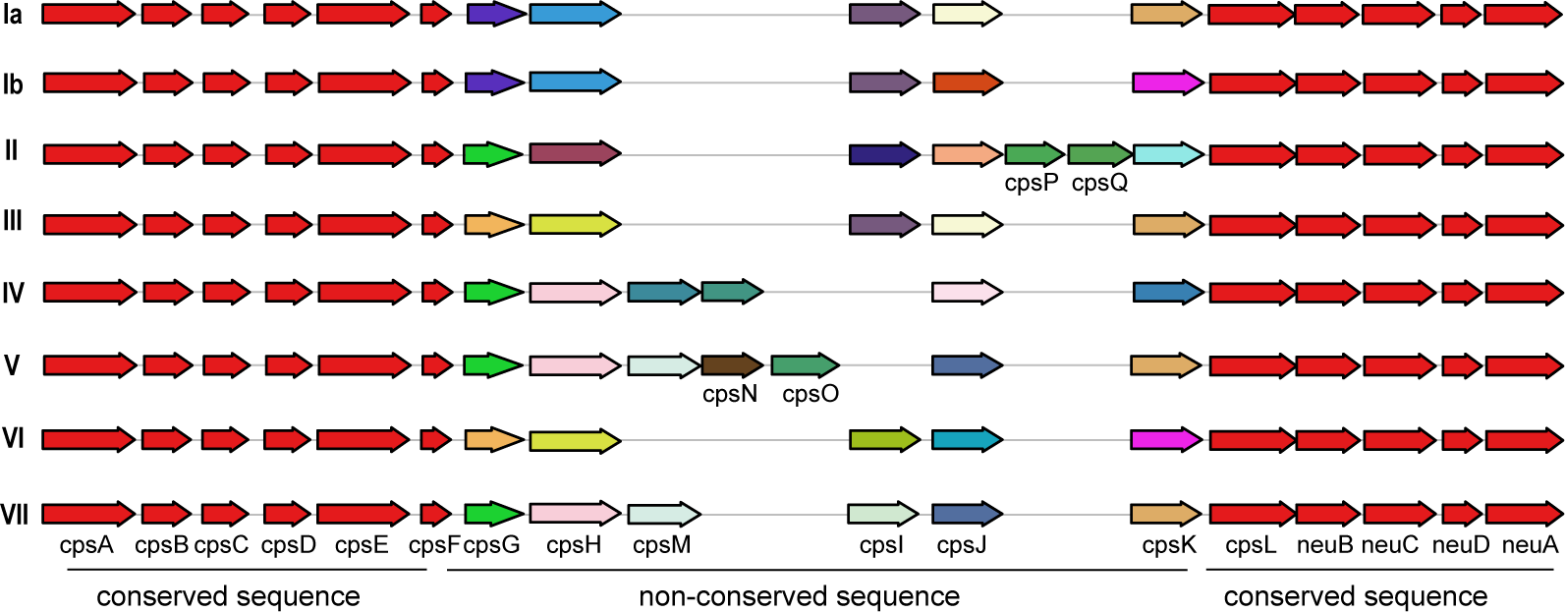
The correlation between the distribution of virulence genes and serotypes and genotypes. This diagram depicts the arrangement of capsular polysaccharide gene clusters in different GBS serotypes (Ia, Ib, II, III, IV, V, and VI). Each row represents a specific serotype, with arrows indicating the direction and identity of individual genes within the CPS gene cluster. Genes that are conserved across all serotypes are shown in red and purple on the left and right ends of each row. Non-conserved sequence: Genes that vary between serotypes are shown in various colors in the middle of each row. The arrangement of these genes highlights the genetic diversity and structural differences in the CPS gene clusters among the GBS serotypes.

Additionally, a comparison between human and non-human hosts revealed disparities in the prevalence of GBS virulence genes. The genes *fbsB*, *hvgA*, *lmb*, *scpB*, and *ssr2* were significantly more common in strains from human hosts, with distribution frequencies exceeding ten folds those in non-human strains. Interestingly, the cyl gene cluster is universally present in genomes from human sources, whereas 30% of genomes from non-human sources lacked this gene cluster. Among these non-human sources, 74% were from fish hosts. A notable finding is that 86% of the GBS strains harbored a single ALP family protein-coding gene, with *bca*, *rib*, and *alp1* being the most prevalent. Notably, the distribution ratio of *rib* is higher in human GBS than in non-human GBS, with a discrepancy of more than nine-fold. Moreover, *alp4* was predominantly found in non-human GBS. The composition of capsular polysaccharides (CPS) forms the basis for serotyping and represents a significant type of virulence factor. An analysis of the CPS-encoding gene composition across different serotypes -Ia, Ib, II, III, IV, V, VI, and VII - presented in **Figure 5**, reveals that genes *cpsA*, *cpsB*, *cpsC*,*cpsD*, *cpsE*,*cpsF*, *cpsL*, *neuB*, *neuC*, *neuD*, and *neuA* were found and conserved in each GBS serotype. Although the genes*cpsG*, *cpsH*, *cpsJ*, and *cpsK* were also present in various serotypes, their sequences were not conserved. The gene *cpsM* was found in serotypes IV, V, and VII, but its sequences were not conserved. Similarly, *cpsN* was found in serotypes IV and V, and its sequences weren’t conserved. The genes *cpsP* and *cpsQ* were unique to serotype II, and *cpsO* was only found in serotype V.

### 3.3 Association of virulence genes with genotypes

**Figure 6:**
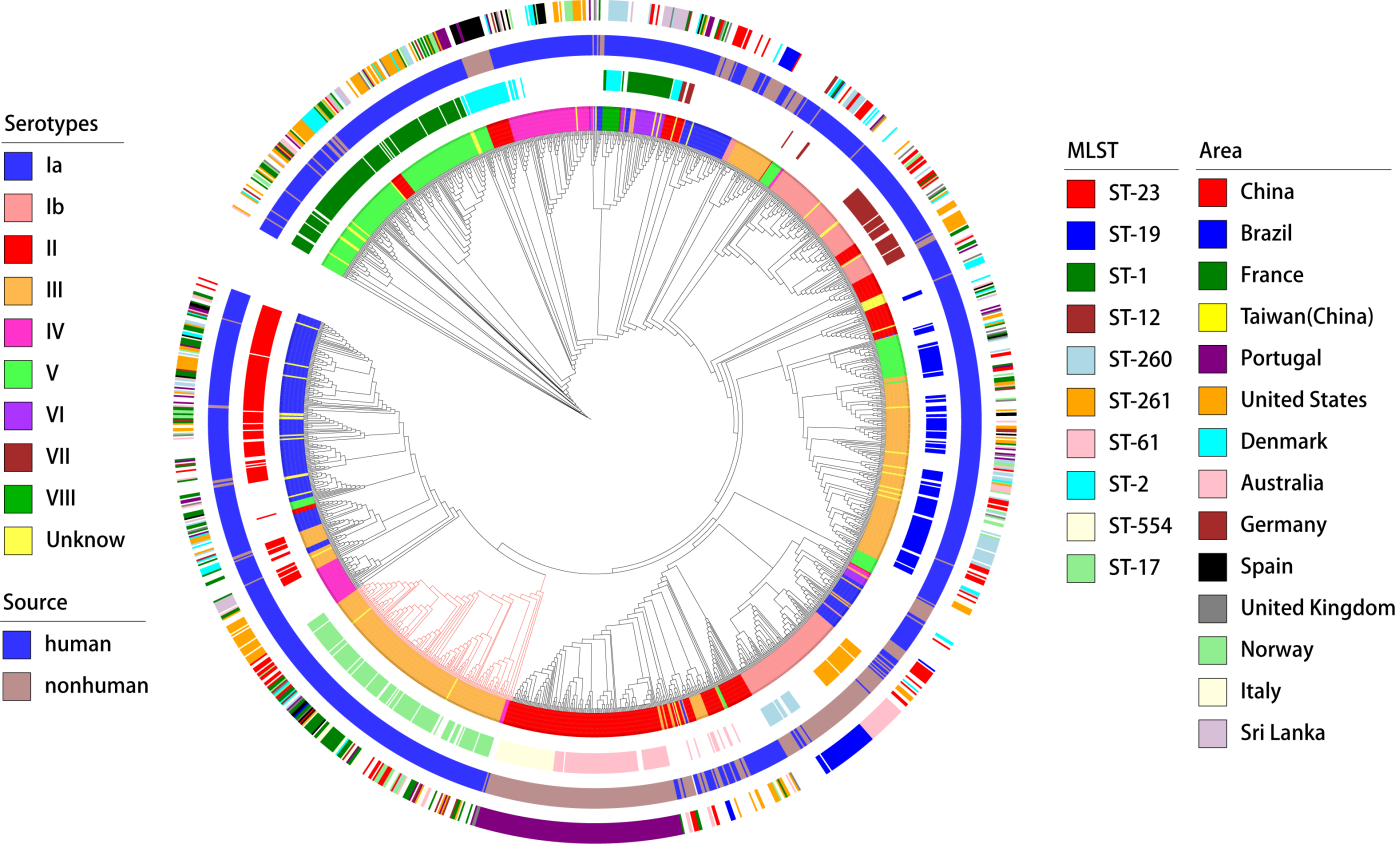
Phylogenetic tree and distribution of GBS strains based on serotypes, sources, sequence types, and geographical areas. This circular phylogenetic tree illustrates the genetic relationships among the GBS strains analyzed in this study. The tree is annotated with multiple concentric rings representing various attributes. Innermost ring (Serotypes): Different colors represent the serotypes of the GBS strains (Ia, Ib, II, III, IV, V, VI, VII, VIII, and Unknown);Second ring (MLST): Multi-locus sequence typing is shown with distinct colors representing different sequence types (e.g., ST-23, ST-19, ST-1, ST-17, ST-54, ST-261, and others);Third ring (Source): The source of the GBS strains is indicated by color, with blue representing human sources and green representing non-human sources; Outermost ring (Area): Geographical regions from which the GBS strains were isolated are indicated by different colors, including China, Brazil, France, Taiwan (China), Portugal, United States, Denmark, Australia, Germany, Spain, United Kingdom, Norway, Italy, and Sri Lanka. The phylogenetic tree provides a comprehensive overview of the genetic diversity and distribution of GBS strains across different serotypes, sources, sequence types, and geographical areas.

A deeper analysis of the connection between the distribution of virulence genes, serotypes, and genotypes was conducted (as shown in the **Figure 6**). The study found marked differences in the annotations of certain genes in strains from human and non-human hosts. The annotation results of GBS virulence genes in human hosts seemed to significantly outnumber those from non-human hosts. The genes *lmb* and *scpB* did not appear to be present in non-human GBS. It’s particularly noteworthy that in ST-17 GBS, the distribution of *hvgA* and *srr2* was clearly specific to this sequence type. Furthermore, the genes *cba* and *bca*appeared to be more readily annotated concurrently in the GBS genome, yet these strains lacked noticeable consistency in the distribution of serotypes or genotypes.

### 3.4 NAAT for GBS screening

Among the 43 patients, the NAAT was positive in 15 cases (34.9%), while the culture method was positive in 3 cases (7.0%). Both methods were positive in 3 cases, and the NAAT was positive but the culture method negative in 12 cases. Notably, GBS was not detected by the culture method in one patient; however, the NAAT was positive, and the newborn was subsequently transferred to the pediatric department due to infection. In total, 3 out of 43 newborns were transferred to the pediatric department.

### 3.5 Identification of potential host factors interacting with Srr1 and Srr2

**Figure 7:**
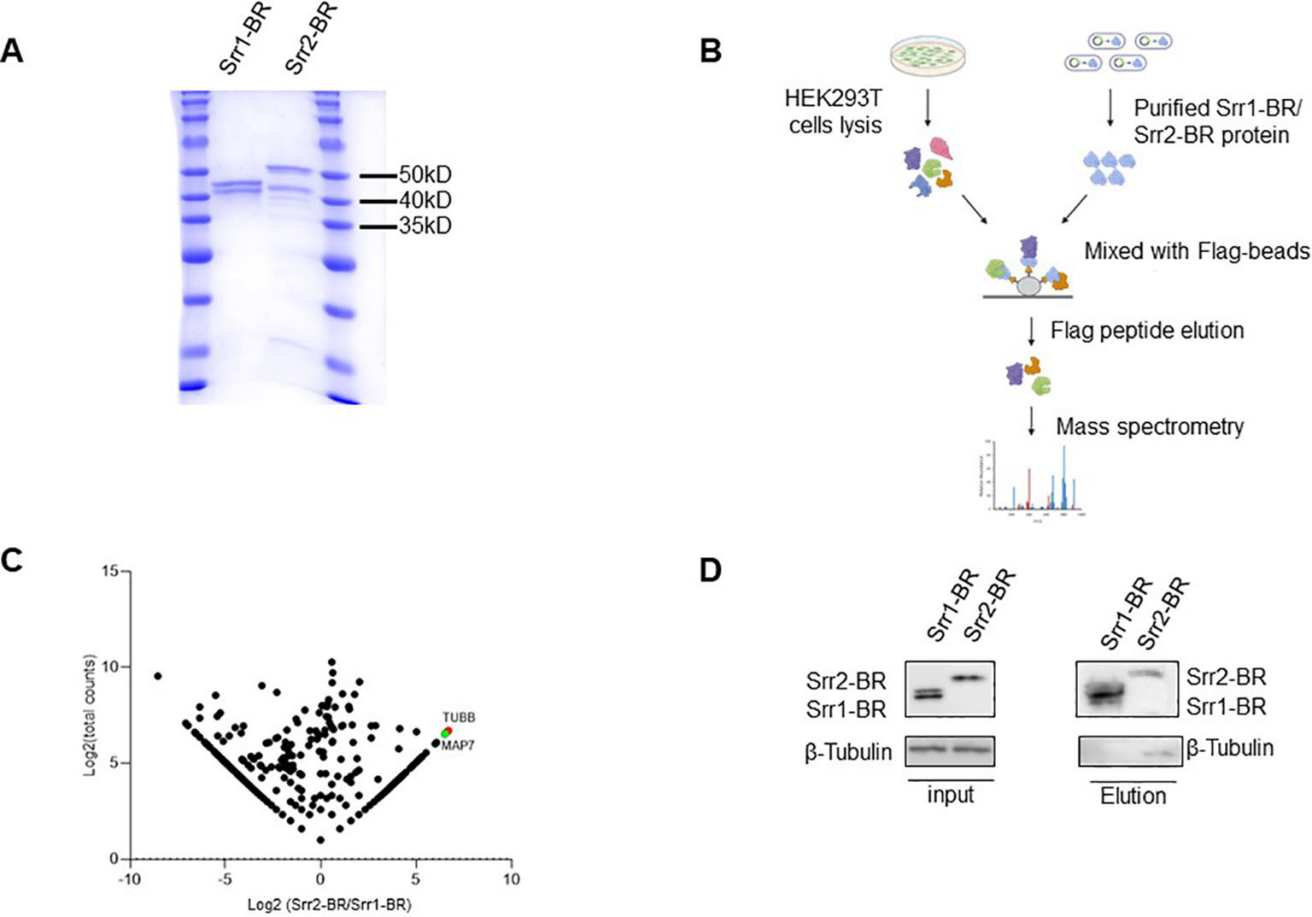
Identification of host factors interacting with GBS Srr1-BR and Srr2-BR. **(A)** Purification of GBS Srr1-BR and Srr2-BR. **(B)** Schematic diagram of workflow to identify host factors interacting with Srr1 and Srr2. **(C)** Comparison of Srr1-BR and Srr2-BR binding proteins identified by mass spectrometry. **(D)** Identification of Tubulin as a Srr2-BR specially binding protein.

In order to explore host factors responsible for the hypervirulence of GBS strains with Srr2 compared to those with Srr1, we identified and compared potential host proteins interacting with them. To achieve this, we expressed and purified the binding regions of Srr1 (Srr1-BR) and Srr2 (Srr2-BR) (**Figure 7A**), which have been demonstrated to response for dock, lock, and latch (DLL) mechanism for GBS-host binding (Seo et al., 2013). Afterward, the HEK293T cell lysate was subjected to incubate with purified Srr1-BR or Srr2-BR, followed by affinity purification and identification through mass spectrometry (LC-MS/MS) analysis (**Figure 7B**).

As shown in **Figure 7C**, the comparison of Srr1-BR and Srr2-BR binding host proteins was conducted using a previously described method (Fu et al., 2024). There are 202 and 411 proteins specifically enriched by Srr2-BR and Srr1-BR, respectively. Among the proteins specifically interacting with Srr2-BR, Tubulin, a microtubule cytoskeleton protein, exhibited the most distinct and abundant interaction with Srr2-BR compared to Srr1-BR. To further validate the interaction between Tubulin and Srr2-BR, elution containing Srr1-BR or Srr2-BR binding proteins as in **Figure 7B** was subjected to immunoblotting with antibody against Tubulin. Notably, cellular endogenous Tubulin was found to specifically interact with Srr2-BR, whereas there was no binding of Tubulin and Srr1-BR (**Figure 7D**), suggesting that microtubule cytoskeleton Tubulin binding ability is Srr2-BR specific. Cytoskeleton rearrangement triggered by bacteria was essential for its phagocytosis and pathogenesis, this distinction of Tubulin binding affinity observed between Srr2-BR and Srr1-BR may provide an explanation for the hypervirulence induced specifically by Srr2-BR.

## 4 Discussion

GBS is the only microorganism specifically targeted for screening in the third trimester, underscoring its significant risk to newborn health. However, GBS, as an opportunistic pathogen, can also colonize the intestinal tract and reproductive tract of healthy people, indicating that the GBS pathogenicity may be affected by host immunity, and may also be related to pathogenicity differences at the strain level. Compared with culture-based screening, the high sensitivity of molecular methods results in a higher positive rate. In previous studies, the colonization rate of GBS in late pregnancy women has been reported to range from 11-35% (Furfaro et al., 2018).In our results, the detection rate of NAAT was higher (34.9%), approaching the upper limit reported in the literature. However, the detection rate of selective culture medium was significantly lower than the lower limit (7%) reported in the literature. These differences indicate that current GBS screening methods require improvement. We hope to find a method that can immediately detect the colonization status of the GBS, and can accurately identify those with high virulence. Specific patterns of GBS virulence gene combinations seem to be a solution to make NAAT tests more targeted on a fundamental basis.

This study confirmed that although certain MLST-based genotypes of GBS exhibit homogeneity in serotypes, there is no overall significant relationship correlation between them. The serotypes of GBS are, in fact, a reflection of the composition of capsular polysaccharide virulence factors. However, previous epidemiological research has shown that both serotypes and genotypes do not provide a good indication of the incidence of clinical GBS and its relationship with neonatal infection. Therefore, we analyzed the composition of virulence genes in GBS and compared them with serotypes and genotypes. It was found that GBS strains derived from human sources tend to possess a greater number of virulence factors. There is a great difference between human and non-human GBS, while the GBS classification features of human are not obvious in the world, this is simply because that the distribution of GBS in the population is not affected by the region. This phenomenon may be due to the widespread nature of human GBS strains and their ability to colonize diverse populations regardless of regional differences. The concentration of non-human GBS features is also observed, potentially attributed to the relatively small number of researchers uploading non-human GBS, and the lack of migration and communication among host populations.

GBS is an important pathogen threatening maternal and fetal health in obstetrics, in which ST-17 strains (capsular serotype III) is considered to be the most virulent and harmful strain. In our study, it was also confirmed that the simultaneous existence of *srr2* and *hygA*, which are highly virulent strain. The GBS Srr family of glycoproteins as surface-associated fibrinogen binding proteins (Fbs) binds to a single tandem repeat region of human fibrinogen via a ‘lock, dock and latch’ mechanism (Seo et al., 2013). The large locus encoding Srr1 and Srr2 are located in different chromosomal locations, but their Srr1 and Srr2 exhibit structural similarity with similar genetic organization. Srr1, which binds to fibrinogen and keratin 4 and thereby mediates adhesion to the vaginal and cervical epithelium, while Srr2 can bind plasminogen and plasmin, which is the characteristic surface glycoprotein of ST-17 strains (Six et al., 2015). In most cases of our study, the *ssr1* and *ssr2* genes are not simultaneously present in a single GBS strain. Only in a few instances are both genes either missing or present together. Highly virulent GBS can cause serious infection outcomes, and the different virulence genes play a series of roles in colonization and invasion at different stages. There may be a potential correlation between different virulence genes, which deserves further study. To investigate the mechanism driving the hypervirulence induced by the concurrent presence srr2 and hygA, we attempted to perform biological experiments aimed at unraveling the molecular pathways and host regulatory factors involved in their functionality. However, purification of hygA proved unsuccessful. Thus, we adopted a compromise strategy to compare the host factors enriched by Srr2-BR and Srr1-BR. The lack of co-existence between Srr2-BR and Srr1-BR, along with the increased virulence of GBS strains carrying Srr2, highlights the importance of comparing host factors that interact with these two variants, providing valuable insights for their distinction. Here, by conducting mass spectrometry analysis, microtubule cytoskeleton Tubulin was found to be the most significant factor interacting with Srr2-BR, and their interaction was further validated by immunoblotting. Cytoskeleton rearrangement represents a commonly employed strategy by diverse pathogens to facilitate their life cycle and pathogenesis (Dramsi and Cossart, 1998; Guiney and Lesnick, 2005). For GBS, it has been shown that cytoskeleton rearrangement is complicated for its invasion (Shin et al., 2006). Given the crucial role of Tubulin in microtubule dynamics and cellular fate, discerning its specific binding preference for Srr2-BR rather than Srr1-BR may offer insights into their distinct virulence mechanisms. In addition, microtubule-associated protein 7 (MAP7), a cofactor for microtubule-associated transport (Ferro et al., 2022) and has been proved to be novel host binding partners factors of pathogenic Escherichia coli (Law et al., 2016), was also among the most abundant interactors of Srr2-BR. Collectively, our findings suggested that hijacking of microtubes and their associated proteins may be involved in the hypervirulence of Srr2.

FbsA, FbsB, and FbsC are three members of the family of fibrinogen binding proteins encoded by GBS, and they adhere to human epithelial cells to promote vaginal colonization (Buscetta et al., 2014).It is reported that FbsC was not expressed in those clinical GBS isolates belonging to the highly pathogenic lineage ST17. But according to our results, all GBS strains poses a FbsC protein (Tazi et al., 2010). HvgA is currently considered to be the most relevant virulence gene for neonatal injury. The GBS hypervirulent adhesin (HvgA) is a novel cell wall anchored protein that is specific for the hypervirulent clone ST-17. It was initially described (Tazi et al., 2010) as being strongly associated with ST-17 causing neonatal meningitis in LOD. It was suggested to promote meningeal tropism in neonates through efficient intestinal colonization and subsequent translocation across the intestinal and the blood brain barriers. The strains isolated from pregnant women were found to encode surface protein virulence factors including HvgA, which helps it to acquire high virulence (Awwad et al., 2022). The specific combination of virulence factors predicts the high virulence of GBS. Highly virulent GBS can cause serious infection outcomes, and the different virulence genes play a series of roles in colonization and invasion at different stages. There may be a potential correlations between different virulence genes, which deserves further study.

In addition to *hvgA* and *srr2*, *cba* and *fbsB* may also be important virulence factors, one is because their prevalenceis relatively low, and the other is because the GBS serotypes corresponding to these two genes can better include types Ia and Ib. This corresponds to the presence of Ia and Ib in GBS isolated from newborns in addition to serotype III in clinical practice. The β protein encoded by *cba* is able to bind to the Fc of host IgA, and complement factor H (FH) (Pleass et al., 2001). The deposition of C3b suggests that the β protein plays an important role in GBS evading host immune system attack (Morgan et al., 1999).

Alpha-like proteins in Streptococcus agalactiae are multifunctional surface proteins that play critical roles in adherence, immune evasion, antigenic variation, and virulence, contributing significantly to the pathogenesis of GBS infections. Alps are immunogenic, give rise to protective antibodies, and are potential vaccine candidates (Stalhammar-Carlemalm et al., 1993; Gravekamp et al., 1997; Kling et al., 1997; Stalhammar-Carlemalm et al., 2007). Hundreds of human GBS strains from various geographical areas have been tested for alp4 possession, but only very few seems to be the only isolate that possesses alp4, the alp4-encoding gene (Kong et al., 2002; Moyo et al., 2002; Mavenyengwa et al., 2008; Maeland et al., 2015).In our study the GBS that detected alp-4 were all non-human sources. Based on antiserum against a serotype III/Rib GBS strain and antiserum against purified Rib, immunological testing proved that Rib is specific (Maeland et al., 2005)and in our result, the presence of rib had high coincidence with the highly independent group carrying hygA and srr2, which suggested that rib was of great significance for vaccine preparation.

In fact, the biological effects of GBS virulence factors in the treatment of GBS are not fully understood. The CAMP factor encoded by the *cfb* is a pore-forming toxin, but it is currently believed not to be essential for GBS pathogenicity. The FbsC protein has been shown to promote GBS adhesion and invasion of epithelial cells and endothelial cells. SfbA can bind to fibronectin, facilitating GBS binding to and invasion of human brain microvascular endothelial cells. The HylB protein of GBS degrades host proinflammatory hyaluronic acid fragments into disaccharide components and exerts immunosuppressive effects by binding to TLR2/TLR4 receptors. GBS lacking HylB exhibits reduced capacity for ascending from the vagina to the uterus. Whether the presence of these four genes (*cfb*, *fbsC*, *sfbA*, and *hylB*) in all GBS strains constitutes the fundamental pathogenicity of GBS at the species level still requires further confirmation.

In summary, the analysis of published typing and virulence gene annotations of GBS genomes revealed no significant correlation between serotypes and genotypes. In contrast, the virulence genes of GBS strains with different phylogenetic relationships showed certain patterns. In future clinical practice, greater emphasis should be placed on the detection of virulence genes to achieve precise GBS surveillance and disease prevention.

## Data Availability

The original contributions presented in the study are included in the article/supplementary material. Further inquiries can be directed to the corresponding authors.
